# Nutrition in acute renal failure

**DOI:** 10.1590/S1516-31802005000300011

**Published:** 2005-05-02

**Authors:** José Paulo Cipullo, Suzana Margareth Ajeje Lobo, Emmanuel de Almeida Burdmann

**Keywords:** Acute kidney failure, Nutrition, Nutritional assessment, Nutrition therapy, Mortality, Insuficiência renal aguda, Nutrição, Avaliação nutricional, Terapia nutricional, Mortalidade

## Abstract

Nutritional status has been considered to be one of the possible determinants of mortality rates in cases of acute renal failure (ARF). However, most studies evaluating possible mortality indicators in ARF cases have not focused on the nutritional status, possibly because of the difficulties involved in assessing the nutritional status of critically ill patients. Although the traditional methods for assessing nutritional status are used for ARF patients, they are not the best choice in this population. The use of nutritional support for these patients has produced conflicting results regarding morbidity and mortality. This review covers the mechanisms and indicators of malnutrition in ARF cases and the types of nutritional support that may be used.

## RESUMO

O estado nutricional tem sido considerado como um dos possíveis determinantes da taxa de mortalidade em insuficiência renal aguda. No entanto, na maioria dos estudos que avaliam possíveis preditores de mortalidade na insuficiência renal aguda, pouca atenção tem sido dada ao estado nutricional, possivelmente em função das dificuldades de sua avaliação em pacientes críticos. Embora os métodos tradicionais de avaliação nutricional sejam usados na insuficiência renal aguda, estes não são os mais indicados para esta população de pacientes. O uso de suporte nutricional nestes pacientes tem originado resultados conflitantes em relação à morbidade e à mortalidade. Esta revisão aborda os mecanismos e marcadores de desnutrição na insuficiência renal aguda e os possíveis procedimentos de suporte nutricional a serem realizados.

## INTRODUCTION

Approximately 5% of a hospital's patient population may develop acute renal failure (ARF).^1,2^ This prevalence is even greater among patients in intensive care units (ICUs), where it may reach 16%.^3,4^ ARF is a severe disease associated with signifi cant mortality rates. In the absence of comorbidity factors, the mortality rate ranges from 7 to 23%, whereas in large series it is around 50%.^2,5^ Several factors have been correlated with this extremely high mortality rate, such as sepsis, multiple organ failure and oliguria.^5-8^

The patient's nutritional status has also been considered to be a possible mortality factor in ARF cases. Although malnutrition is highly prevalent among hospitalized patients and is associated with increased mortality,^9-11^ the current literature on nutrition in ARF cases is fundamentally based on experimental studies. Most of the clinical studies available have been based on small numbers of patients, retrospectively analyzing surgical or intensive care units (ICU) populations.^12-17^ The sampling problems may result from difficulties in assessing the nutritional status of critically ill patients.

One of the most important initial studies, aimed at evaluating the effect of nutritional therapy in ARF cases was carried out by Abel et al. (1973).^12^ These authors reported lower mortality among ARF patients receiving a combination of essential amino acids and hypertonic glucose, in comparison with patients receiving hypertonic glucose alone. Subsequently, several investigators evaluated the role of nutritional supplementation in morbidity and mortality among ARF patients and obtained confl icting results.^13-17^ These studies were the subject of a meta-analysis carried out by Naylor et al. (1987-88).^18^ This meta-analysis concluded that the survival of ARF patients receiving parenteral nutrition with essential amino acids and hypertonic glucose solutions was better than for those receiving hypertonic glucose alone. However, most of the studies examined had poor descriptions of the randomization criteria and inappropriate statistical analysis, and only two of the four randomized studies included more than 10 patients in each study group. Thus, the authors of the meta-analysis concluded that the effi cacy of parenteral nutrition regimens in ARF cases remains unclear.

### Energy metabolism

Energy expenditure is defi ned better in relation to the underlying disease than to acute uremic status, thus suggesting that, when uremia is well controlled, there is little variation in energy metabolism.^19-21^ Energy requirements may be accurately calculated using the Harris-Benedict equation multiplied by the associated stress factor.^22^ Even under hypercatabolic conditions, such as sepsis or organ dysfunction, the energy requirements rarely exceed 1.3 times the baseline energy expenditure.^22^ In ARF cases, an energy intake of 25 to 35 kcal/kg/day is recommended, according to the associated degree of catabolism.^22^

### Protein metabolism

The most marked alteration in nutritional status among ARF patients is the presence of hypercatabolism with a negative nitrogen balance.^23^ Several factors may contribute towards the increased catabolism in ARF cases. Infl ammatory mediators, including interleukins and tumoral necrosis factor, activate the protein metabolism in the same way as in other conditions like sepsis that are observed among critically ill patients. Data obtained from animal studies suggest that uremia is associated with increased gluconeogenesis, with increased protein catabolism and reduced protein synthesis.^24^ Hormonal and metabolic changes, such as insulin resistance, increased glucagon concentrations, secondary hyperparathyroidism and metabolic acidosis have also been correlated with malnutrition among ARF patients.^25,26^

Dialysis procedures cause nutrient loss and stimulate protein catabolism. In low-flux membrane dialysis, 5 to 8 g of amino acids are lost per session. When high-flux membranes are used, these losses may increase by 30%.^27,28^ The structural characteristics of the dialysis membrane may also influence the nutritional status. The lower the membrane biocompatibility is, the greater the cytokine release related to increased protein catabolism will be, thereby delaying the recovery of ARF and producing worse outcomes.^29,30^The amount of dialysis may also affect the nutritional status. It is well known that inadequate dialysis doses are associated with worsening of the nutritional status of patients with chronic renal failure.^31^ It has been reported that the delivered dialysis dose in patients with ARF is frequently lower than the prescribed dose,^32,33^ and that uremia induced by suboptimal dialysis is likely to aggravate the protein catabolism.

In experimental ARF models, in addition to increased proteolysis, reduced protein synthesis is also observed. In muscles, the protein degradation rate is increased at the same time as protein synthesis is reduced, even in the presence of insulin.^24^The inability to improve the nutritional parameters in most ARF patients, despite adequate nutritional therapy, is probably caused by failure to uptake and use the available nutrients. There is evidence that uremia causes abnormalities in the growth hormone/insulin-like growth factor-1 axis and induces resistance to the activity of the growth hormone at a cellular level, thereby hindering the optimal uptake of nutrients.^34^ Concomitant diseases and conditions frequently associated with ARF, such as sepsis and organ failure, also reduce anabolism.

### Nutritional assessment in acute renal failure

Several nutritional assessment methods have been suggested, using clinical, biochemical, anthropometric and body component evaluations. However, no single indicator may be considered to be a "gold standard". Many of the traditional nutritional evaluation methods used in ARF cases are affected by non-nutritional factors, and therefore ought not to be used in this patient population. The most important reason for discrepancies is that several processes play a role in the malnutrition of hypercatabolic patients with ARF. In these patients, malnutrition is a metabolic response to stress or inflammation, whereas under other conditions, malnutrition is predominantly a response to chronic inanition. Nutritional marker variations might differ considerably under these two conditions.^32,35^

#### Insulin-like growth factor-1

Insulin-like growth factor-1 (IGF-1) and insulin-like growth factor-2 (IGF-2) are structurally related to insulin. Human IGFs, generically called somatomedins, are simple chain peptides of 7.5 kilodaltons (kDa), composed of 70 (IGF- 1) or 67 amino acids (IGF-2).^36^Four properties, designated A, B, C and D, are identified in IGF molecules, and the A and B properties are homologues for the insulin A and B chains. Somatomedins are secreted at the same time as they are produced, and consequently they are not concentrated in any organ. Therefore, although the liver is the major source of circulating somatomedins,^37^ their highest concentration is observed in the blood.^38^ IGFs are produced in several organs and are biologically active in most cell types.^39^ These peptides act through autocrine and paracrine mechanisms, as well as through the classic endocrine mechanisms.^40^The growth hormone is one of the major hormonal stimuli for IGF-1 production.^41^Less than 5% of circulating IGF-1 is free, and over 90% is tied to other binding proteins.^42^ IGF-1 synthesis is influenced by hormonal and nutritional factors.^43^ In humans, serum IGF-1 levels are reduced after protein-energy deprivation, and return to normal levels within few days after food intake is resumed.^44^ IGF-1 is a reliable marker for nutritional status and has been shown to be better than other biochemical markers for assessing nitrogen balance in severely ill and hypercatabolic patients.^45^ Unterman et al. (1985)^46^ reported that, in malnourished patients, IGF-1 serum levels were better indicators than laboratory tests and the classic anthropometric parameters used to evaluate nutritional status. Donahue and Phillips (1989)^47^ showed that decreased IGF-1 serum levels in hospitalized patients with protein or protein-energy malnutrition (39 ± 7 ng/ml) were more marked than in patients with energy malnutrition alone (109 ± 25 ng/ml), thus confirming the major role of protein intake in IGF-1 regulation. The good correlation of IGF-1 with nutrient deprivation or intake, its serum stability and short half-life recommend its use as a marker for nutritional status in ARF cases.^42,45,48^

#### Albumin

Serum albumin levels decrease markedly in response to stress and inflammation,^49^ and may not accurately reflect nutritional status changes in severely ill patients. The serum half-life of albumin is relatively long (20 days) and serum albumin concentrations change in response to catabolism and nutritional supplementation that take place in the late-stage course of acute diseases.^50^ Hypoalbuminemia has been described as an independent factor for mortality among elderly patients and patients with chronic renal failure undergoing dialysis.^51,52^ However, there is little evidence for an association between decreased serum levels of albumin and mortality in ARF cases. Decreased serum albumin levels have been described by Chertow et al. (1998)^6^ as predictors of mortality among patients with acute tubular necrosis. It should be emphasized that, up to that time, there had not been any reports in the literature to associate hypoalbuminemia with higher mortality in ARF patients. In a study of 15,000 severely ill patients, serum albumin levels < 3.4 g/dl on admission were strongly associated with higher mortality, prolonged hospitalization and readmission.^52^

#### Transferrin

Transferrin has a shorter half-life (8 days) than albumin, but it lacks sensitivity for evaluating the short-term effects of refeeding.^53^ Transferrin concentration is significantly influenced by patients' serum iron levels.^54^

#### Prealbumin

Serum prealbumin is a nutritional marker with a half-life of 1 to 2 days and a good response to nutritional supplementation.^53^ Similarly to albumin, its serum levels decrease in response to stress and inflammation.^49^ It is excreted mainly by the kidneys. Prealbumin concentrations may be falsely high in patients with ARF.^55^

#### Body composition analysis

The standard techniques for assessing the different body compartments in hospitalized patients were proposed by Blackburn et al. (1977).^56^ Fat storage and the quantification of somatic proteins are usually evaluated by anthropometric measurements. Although these techniques are simple, safe and extensively used to assess body composition in different types of population, they do not have a good clinical correlation in individual analyses.^54^ The use of these methods in ARF cases has limited value, due to the frequent fluid variations observed in this type of patients. Likewise, bioelectrical impedance analysis is a noninvasive, fast, sensitive and accurate method for body composition measurement.^55^ However, its use in ARF cases is also limited due to fluid variations.

#### Subjective global assessment

Subjective global assessment (SGA) is a technique used for evaluating nutritional status. It assesses the nutritional status based on clinical experience and includes the medical and nutritional history, physical examination and functional assessment of the patients. This method was initially developed to assess the nutritional status of surgical patients,^57,58^ but it has now been used in several groups of patients.^59,60^ In patients with chronic renal failure, good correlation has been found between diagnoses of malnutrition made via SGA and objective methods, including biochemistry tests and body composition measurement.^59,61^ Abdullah et al. (1998)^62^ found lower levels of anabolic factors, such as IGF-1, and higher catabolic cytokine levels in patients classified as malnourished via SGA. Worsening of nutritional status, as evaluated by the SGA method in chronic renal patients undergoing peritoneal dialysis, has been correlated with greater mortality risk,^63^ although the results from subsequent studies have conflicted with this.^64,65^ Fiaccadori et al. (1999)^66^ reported that nutritional status changes, as assessed by the SGA method, are frequent findings among ARF patients. The same authors reported that preexisting malnutrition, characterized by SGA class C, was an independent predictor for hospital mortality.

Since SGA is a subjective technique, it does not measure visceral proteins and does not provide follow-up for nutritional therapy. Moreover, because it requires information from patients, this methodology may not always be applicable to ARF patients in the ICU, due to their decreased consciousness levels caused by their underlying disease or by the use of sedatives, or because of the use of mechanical ventilation.

#### Total cholesterol

Serum cholesterol is an independent predictor for mortality among hemodialysis patients. Individuals undergoing hemodialysis with normal or low (from 150 to 180 mg/dl) serum cholesterol levels have higher mortality than those with higher cholesterol levels.^67,68^ However, the association between hypocholesterolemia and mortality due to non-cardiovascular causes is unclear.^69^ Hypocholesterolemia and decreased low density lipoprotein (LDL) have been described in severely ill surgical patients in the ICU with evidence of sepsis.^70^ It is not clear under what circumstances serum cholesterol might be a reliable indicator of protein-energy malnutrition. Additional data on the associations between serum cholesterol, nutritional status and morbidity and mortality are required.

### Patient classification and nutritional therapy

The use of adequate nutritional therapy among patients suffering from different diseases is required in order to maintain protein storage and regulate lean body mass deficits. The objectives of nutritional therapy among ARF patients are no different from those under other hypercatabolic conditions. However, they are different from the objectives to be achieved among patients with chronic renal failure (CRF), since therapies meeting the minimum requirements for CRF are insufficient for ARF patients.^22^

Since not all ARF patients necessarily require nutritional support, it is important to identify those who will benefit from it, as well as to establish the optimal time to start therapy. The decision to start therapy is influenced by the individual's capacity to adequately intake the nutritional requirements, by nutritional status and by the type of underlying disease. When there is evidence of malnutrition or hypercatabolism, therapy should be started early on.

Nutritional requirements are often neglected in clinical practice. Urea nitrogen appearance rate (UNA) measurements, which reflect protein catabolism,^22^ and the assessment of energy requirements are not routine practice. The formulae usually used to calculate energy requirements may underestimate these requirements among ARF patients, since they are based on healthy individuals with normal body fluid distribution.

The undesirable effects of nutritional therapy are another limitation on its use. Excessive supplementation of proteins increases the end products of protein metabolism. The provision of large amounts of nutrients requires the infusion of considerable quantities of fluids, carbohydrates and lipids, and this may cause volume overload and undesirable electrolytic and metabolic changes, such as hyperglycemia, hyperlipidemia, hypernatremia or hyponatremia. Although most of these changes may be controlled by dialysis, the possibility of such changes induces physicians to use more conservative approaches towards nutritional therapy, thereby inadvertently contributing to a worsening of the nutritional status of ARF patients.

Nutritional programs must be individually designed for each ARF patient. In clinical practice, patients may be divided into three groups,^22^ according to the degree of catabolism, which may be evaluated by calculating the UNA rate (Table 1).

**Table 1:** Estimating the extent of protein catabolism

Urea nitrogen appearance (UNA) (g/day) = urinary urea nitrogen excretion + change in urea nitrogen pool = (UUN × V) + (BUN_2_ – BUN_1_) 0.006 × BW + (BW_2_ – BW_1_) × BUN_2_/100

If there are substantial gastrointestinal losses, add urea nitrogen in secretions:
= volume of secretions × BUN_2_
Net protein breakdown (g/day) = UNA × 6.25
Muscle loss (g/day) = UNA × 6.25 × 5

*UUN = urinary urea nitrogen concentration in grams nitrogen/day; V = urinary volume in liters; BUN1 and BUN2 = blood urea nitrogen in mg nitrogen/dl on days 1 and 2; BW1 and BW2 = body weights in kg on days 1 and 2.*

GROUP I: Low UNA rate. These patients are mildly catabolic, i.e. those whose ARF was caused by nephrotoxins alone (aminoglycosides, contrast media and others). Dialysis is seldom required and the use of nutritional therapy containing 25 kcal/kg/day and 0.6 g/kg/day of proteins rich in essential amino acids is usually sufficient. Such patients are usually fed orally and the prognosis for the recovery of renal function and survival is excellent.^22^

GROUP II: Moderate UNA rate. These are ARF patients with moderate catabolism, frequently suffering from infectious or surgical complications. The use of enteral or parenteral nutrition and dialysis is often required. These patients should receive essential and non-essential amino acids at a dose of 0.8 to 1.2 g/kg/day and calorie intake of 25 to 30 kcal/ kg/day. The mortality rate in this population is approximately 60%.^22^

GROUP III: High UNA rate. These are patients who develop ARF in association with severe trauma, severe burn injuries and sepsis. The treatment for this population is complex and includes parenteral nutrition and dialysis. Ventilatory and hemodynamic support are often required. The nutritional requirements for reducing catabolism and minimizing protein depletion are high. The energy requirement is approximately 25 to 35 kcal/kg/day and the protein requirement is 1.0 to 1.5 g/kg/day. The mortality rate in this group is greater than 80%.^22^

It should be stressed that, in groups II and III, even early and optimized use of nutritional support will hardly be able to offset the marked negative nitrogen balance observed in such patients.

## CONCLUSIONS

Nutritional therapy in ARF patients must include an individualized program to meet the nutritional needs of several degrees of stress and hypercatabolism. In these patients, the major determinants of nutritional requirements are not the ARF itself, but the degree of catabolism of the associated diseases, the nutritional status and the type and frequency of dialysis. If there is evidence of malnutrition or hypercatabolism, the therapy should be started early. So far, there is no clear evidence that any specific type of nutritional support is capable of changing the natural history of ARF.

The methods for evaluating the nutritional status and short-term nutritional changes among critically ill patients are insensitive (albumin and anthropometric parameters), have low specificity (transferrin and prealbumin) or are difficult to carry out (nitrogen balance). A sensitive, specific and easy-to-measure marker is clearly required for the early diagnosis of malnutrition in ARF.
